# Measles-mumps-rubella-vaccination at 6 months of age induces measles-specific T cell responses: a randomized controlled trial

**DOI:** 10.3389/fimmu.2025.1546253

**Published:** 2025-03-17

**Authors:** Søren Buus, Dorthe Maria Vittrup, Jonas Damgård Schmidt, Andreas Jensen, Anette Stryhn, Lone Graff Stensballe

**Affiliations:** ^1^ Department of Immunology and Microbiology, University of Copenhagen, Copenhagen, Denmark; ^2^ The Child and Adolescent Department, The Juliane Marie Center, Copenhagen University Hospital, and Mary Elizabeth Hospital, Rigshospitalet, Copenhagen, Denmark; ^3^ Department of Clinical Medicine, University of Copenhagen, Copenhagen, Denmark

**Keywords:** MMR, measles vaccination, early immunization, immunogenicity, cellular immune responses, double-blind randomized placebo-controlled clinical trial (RCT)

## Abstract

**Background:**

Measles is a highly contagious viral disease, particularly severe in infants. Protection in early life is provided by maternally transferred antibodies, but this period is shorter in infants of previously vaccinated mothers (PVMs) compared to infants of previously measles-infected mothers (PIMs). Earlier measles-mumps-rubella (MMR) vaccination may compensate for this. To evaluate immune responses, 6-month-old infants were randomized to receive early MMR or placebo. This study reports the cellular immune outcomes and summarizes serological and T-cell responses.

**Methods:**

A double-blind, randomized trial involved 6540 Danish infants aged 5–7 months, eligible if birth weight exceeded 1000 grams and gestational age was ≥32 weeks. Participants were randomized 1:1 to receive M-M-RVaxPro or placebo. Blood samples were collected before intervention, four weeks after intervention, and four weeks after routine MMR at 15 months. Peripheral blood mononuclear cells (PBMCs) were prepared, and an IFN-γ specific ELISpot assay measured measles-specific T cells.

**Results:**

Among 750 infants (341 MMR, 409 placebo) in the cellular immunogenicity trial, a significant cellular immune response was observed one-month post-intervention in the MMR group compared to placebo (geometric mean ratio [GMR]: 12.3; 95% CI: 6.9–21.9). The cellular conversion rate (CCR) in the MMR group was 45%, comparable to the previously reported seroconversion rate. However, following routine MMR at 15 months, a reduced cellular response was observed in the early MMR group (GMR: 0.6; 95% CI: 0.3–0.9). Post-routine MMR, CCRs were 66% (MMR) and 74% (placebo). The immune conversion rate (ICR, defined as seroconversion and/or T-cell response) reached 99% in both groups post-routine MMR.

**Conclusion:**

Early MMR at 6 months elicited significant measles-specific cellular responses, though the CCR was lower than after routine MMR at 15 months. However, when combining serological and cellular responses, 99% of infants achieved immune conversion by 15 months. Early MMR could help reduce measles burden in infants in endemic settings without compromising subsequent immunizations.

**Clinical trial registration:**

ClinicalTrials.gov, identifier NCT03780179, EudraCT 2016-001901-18.

## Introduction

Measles is a significant global public health challenge. It is caused by the measles virus, one of the most contagious viruses known (1). In theory, measles could be eradicated if a sufficiently high percentage of the population were immune. However, despite the availability of safe and cost-effective vaccines, measles continues to be a major cause of morbidity and mortality particularly among children where the case fatality ratio ranges from 0.01% to more than 5%, influenced by factors such as vaccine coverage and access to health care (2). The inclusion of measles vaccines in national immunization programs (NIPs) has reduced the incidence and fatality rate of measles by 75% and 82%, respectively (3). The two-dose vaccination schedule recommended by the WHO has resulted in 95% of recipients achieving protective immunity (4). The timing of the first dose is critical. In endemic settings, where early protection is essential, the first dose is recommended at 9 months of age. In high-risk situations such as measles outbreaks, the WHO recommends an early dose of MCV from 6 months of age in addition to the regular two-dose schedule included in most National Immunization Programs (5). Conversely, in low-risk settings, it is advised to administer the first dose from 12 months of age. The latter would give maternal antibodies, which otherwise could inhibit the vaccine’s efficacy, time to wane, thereby ensuring that the vaccine elicits a robust and durable immune response in most of the children.

A concerning trend has emerged: mothers who have received measles-containing vaccines (MCVs) tend to have lower measles antibody levels compared to mothers who have acquired immunity through natural infection (6, 7). Consequently, infants born to vaccinated mothers receive fewer maternal anti-measles antibodies, and become susceptible to measles infection at a younger age than infants born to naturally infected mothers (8). This increases the risk of outbreaks in infants, causing serious and potentially fatal measles infections. This predicament is further exacerbated in areas with low vaccine coverage.

This raises the question of whether this immunity gap can be bridged if the first MCV is administrated earlier. Closing this gap would protect infants at a particularly vulnerable time of life; however, if it led to an impaired primary vaccine response and/or to a faster decay of antibodies it might have detrimental consequences for the duration of clinical protection. Unfortunately, the evidence addressing this question is sparse. To our knowledge, just a few clinical trials have reported on measles immunogenicity following MMR in 6–8-month-old infants (9–15). Although, non-placebo-controlled studies have documented safety of vaccinating 6-8-month-old infants with standard titer MCV (11–13, 16), further investigations of the immunogenicity and safety of early MMR vaccination are warranted.

Since 1987, the Danish NIP has included a two-dose MMR vaccination schedule; currently, the M-M-RVaxPro is administered at 15 months of age and 4 years of age. In 2017 and 2019, Denmark achieved verified elimination status of rubella and measles, respectively (note, the last rubella case in Denmark occurred in 2008). Given the need for more data on the effects of MMR in early infancy in the post-vaccine era, we initiated a double-blind, placebo-controlled randomized controlled trial (RCT), the “Danish MMR trial”, from 2019 to 2022. This study aimed to determine whether moving the primary MMR vaccination forward to 6 months of age would be safe, free from increased reactogenic effects, and capable of inducing specific protective immunity. The trial enrolled 6540 infants in the reactogenicity study and 647 in the serological immunogenicity study. In a previous publication, we have detailed reactogenic and serological findings. In brief, we found that administering an additional early MMR vaccine at 6 months is safe and does not result in higher rates of reactogenicity than placebo; that it results in a 47% seroprotection rate compared to 13% in placebo-infants; and that it does not impair the serological immune response to a subsequent routine MMR vaccination at 15 months of age (10).

The importance of cellular immunity in protecting humans against measles has been inferred from case-studies prior to the introduction of measles vaccines where immunocompromised individuals were likely to contract measles. When patients with B cell deficiencies were measles infected, the infection was resolved, leaving a protective immunity similar to that of immunocompetent individuals. In contrast, in patients with T-cell deficiencies, the infection could take a fatal course (17, 18). Studying the protective mechanisms induced by live attenuated measles vaccines in a non-human primate model, Griffin and coworkers found that whereas antibodies are crucial for preventing measles infection, T cells are critical for controlling and clearing the infection (19–21).

In this report, we present findings from the Danish MMR trial regarding cellular immunity as assessed by ELISpot analysis measuring the frequency of measles-specific T cells in peripheral blood mononuclear cells (PBMCs) four weeks post-vaccination. Further, the total of serological and cellular immune response after MMR-vaccine at 6 months of age is addressed.

## Materials and methods

### The Danish MMR trial

From April 2019 to January 2022, the Danish MMR trial was conducted as a randomized, double-blind, placebo-controlled trial at Copenhagen University Hospital, Rigshospitalet, Denmark, and ended due to completion. The two co-primary outcomes of this trial were humoral immunogenicity measured 3-5 weeks after intervention and potential non-specific effects (measured by hospitalizations for non-target infectious diseases before the age of 12 months) of MMR vaccination at 6 months of age, which, along with an evaluation of the vaccine’s reactogenicity, have been detailed in previous publications (10, 22). A secondary outcome of this trial, the measles-specific cellular immunogenicity of early MMR vaccination, is reported here. The primary analysis used to establish this secondary outcome was to measure the number of measles-specific T cells four weeks after intervention (MMR/placebo) at 6 months of age using an interferon-γ (IFN-γ) specific ELISpot assay.

Two substantial changes occurred during the trial. Firstly, about half a year into the project, the COVID-19 pandemic caused lockdowns and a worldwide shortage of crucial assay reagents and utensils, which forced us to change the ELISpot analysis from one of testing PBMCs fresh *ex vivo* to one of cryofreezing PBMCs for later *in vitro* ELISpot testing. Secondly, due to regulatory concerns, the positive ELISpot control was changed from Staphylococcal enterotoxin B (SEB) to anti-CD3 antibodies (see below).

### Participants

Healthy infants aged 5–7 months, born in the Capital Region of Denmark with a birth weight of at least 1000 g and a gestational age of 32 weeks or more, were eligible for enrollment. Exclusion criteria aligned with contraindications for the routine administration of M-M-RVaxPro. The immunogenicity subpopulation was determined by parental selection rather than by trial staff. Given that the trial was conducted in a setting of measles and rubella elimination and in MMR-naïve individuals, baseline antibodies detected in the infants at the outset, were presumed to be of maternal origin. During the trial, four measles cases, ten mumps cases, and no cases of rubella were found and confirmed in Denmark (10). No participant was given financial incentives. All enrolled families were advised to follow the Danish National Immunization Program (NIP), which recommends MMR vaccinations at 15 months and again at 4 years of age.

### Vaccination, randomization and masking of participants

Infants were individually allocated in a 1:1-ratio to receive an intra-muscular injection in the anterolateral region of the thigh with either M-M-RVaxPro (23) or placebo (vaccine solvent: consisting of sterile water, ensuring identical handling, packaging, and delivery as the actual vaccine). Randomization was initiated after the child examination and was executed within REDCap stratified by sex and prematurity (gestational age <37 weeks), using permuted blocks of 2, 4, or 6 participants. This task was done by a staff member who did not interact with the participants. Using colored tape, the syringe was blinded from both the specially trained staff member administering the injection and the parents. The allocation results were securely stored in REDCap and remained encrypted until the unblinding event, which occurred either after the last randomization or when the participant reached one year of age, whichever occurred last.

### Blood samples and peripheral blood mononuclear cells (PBMC) preparation

Blood samples were collected through cubital venipuncture, which was carried out after the application of local anesthetic band-aids to minimize discomfort. Immediately before the intervention, blood samples were obtained from the mothers and infants. Additional blood samples were obtained from the infants 3 to 5 weeks after the initial intervention and again after the routine MMR vaccination administered at 15 months of age (22). Note that this sampling time point was chosen to optimize the primary outcome, the serological analysis; it was not optimal for secondary outcome, the T cell analysis, which rather would have been 14 days after vaccination (24).

PBMCs from up to 10,5 ml blood samples were isolated through density gradient centrifugation using Bio-One LeucoSep™ polypropylene tubes (Greiner 227290) and Ficoll-Paque™ Plus (GE Healthcare Europe, Brøndby, Denmark). The PBMCs were either examined ex vivo or cryopreserved in 10% DMSO and 90% FBS at −180°C for later *in vitro* analysis. The actual volume of blood and quantity of PBMCs obtained from each infant varied considerably.

### Analysis of the measles-specific cellular immune response

#### Measles vaccine strain and proteome

The measles virus is a single-strand, negative sense RNA virus within the Morbillivirus genus of the Paramyxoviridae family. The M-M-RVaxPro vaccine strain (Merck) contains the Moraten (“More attenuated Enders”) measles vaccine strain developed by Merck from the Enders’ Edmonston measles virus strain (25). The Moraten genome encodes 8 proteins with a total of 5205 amino acids (Genome Accession# AF266287, Proteome Accession# AAF85667 through AAF85674).

#### Measles peptides

We designed an overlapping peptide library that systematically covered the entire proteome of the Moraten vaccine strain. Each peptide was 17 amino acids in length, arranged in a manner tiling by 7 amino acids and overlapping by 10 amino acids. This ensures the inclusion of every conceivable 11-mer sequence from the proteome, which allowed processing and presentation of virtually all measles-specific, HLA class I- or II-restricted peptides. A total of 705 overlapping peptides were required to represent the full proteome of the Moraten vaccine strain (considering that the initial 231 amino acids of the phosphoprotein (AFF85688) and the V protein (AAF85669) share identical sequences). The peptides were synthesized using standard 9-fluorenylmethyloxycarbonyl (FMOC) chemistry and subsequently purified through reversed-phase high-performance liquid chromatography to achieve a minimum purity of 80%, typically exceeding 95%. Each peptide was confirmed by mass spectrometry (Schafer-N, Copenhagen, Denmark). Out of the 705 peptides planned, 658 (93%) were successfully synthesized.

#### Peptide pools

The peptides were grouped into five pools, each containing approximately 130 peptides (pool 1 contained peptides from the nucleoprotein, the V protein, the C protein, and a few of the peptides from the phosphoprotein; pool 2 contained the remaining peptides from the phophoprotein, peptides from the fusion protein, and some from the large protein; pool 3 contained the peptides from the M protein, the Ha protein and some from the large protein, and pools 4 and 5 contained the remaining peptides from the large protein). The peptides were dissolved, pool-wise mixed equimolarly, and re-lyophilized. Before use, the peptide mixtures were dissolved in DMSO and then diluted in media to a concentration of 1.7% DMSO in this intermediary peptide mixture stock, which would lead to a final *in vitro* culture concentration of DMSO of 0,1%).

#### ELISpot analysis

Fresh or thawed PBMCs were tested using an IFN-γ specific ELISpot assay as previously described (26). Depending on the number of PBMCs available, 2–5 × 10^5^ cells/well were plated in two to four wells of a 96-well ELISpot plate (MAHAS4510, Merck Millipore, USA) for each experimental condition. These conditions included testing the five different measles peptide mixtures, alongside a negative control and a positive control, totaling seven unique experimental setups. The cells were incubated *in vitro* for 18–24h at 37°C in a 5% CO_2_ atmosphere, using X-vivo 15 media (Fisher Scientific) supplemented with 5% AB Serum (Invitrogen) and the respective pool mixtures of peptides achieving a final peptide concentration of 0.5 μM each. Median supplemented with mitogens such a Staphylococcal enterotoxin B (SEB, Sigma Aldrich, St. Louis, USA) at 1 μg/ml, or anti-CD3 (mAb CD3-2, Mabtech, Nacka Strand, Sweden) at 0.1 μg/ml, was used to generate positive controls (see also **Supplementary Table S1**); media alone was used to generate negative controls. In all cases, the final DMSO concentration was 0.1%. Secreted IFN-γ was captured with biotinylated anti-human IFN-γ (mAb 7-B6-1, Mabtech), detected by streptavidin conjugated alkaline phosphatase (Streptavidin ALP, Mabtech) and developed by substrate (AP Conjugate substrate, Bio-Rad, Hercules, USA). Analysis was done using ImmunoSpot 5.0.9 software (C.T.L., Shaker Heights, USA), where wells displaying a count of spot-forming units (SFU) greater than twice the background were deemed positive.

### Cellular conversion rate (CCR)

Here, the prevalence of inducing a cellular immune response is denoted as the cellular conversion rate (CCR). We defined the CCR as the proportion of individuals with measles-specific T cell responses exceeding the 95th percentile observed in the overall infant population at baseline, which is expected to be measles naïve (*in casu* > 16 SFU/10^6^ PBMCs). We defined the immune conversion rate (ICR) as the proportion of infants that mounted a humoral and/or cellular immune response following intervention and/or routine MMR.

### Analysis of the measles-specific humoral immune responses

The analysis of the humoral immune responses has been described and reported previously (10). Briefly, serum was prepared from a smaller, 3.5 ml, blood sample and frozen at -80 °C until use. An established measles plaque reduction neutralization test (PRNT) protocol (27) was used to determine the 50% plaque reduction titer, and a commercial ELISA kit (Creative Diagnostics kit number DEIA359) was used to estimate the measles-specific IgG concentration after standardization using a WHO 3rd International Standard for measles antibodies.

### Statistics

The primary outcome of the present report was the level of measles-specific T cells based on ELISpot measured four weeks after intervention (MMR/placebo) at 5-7 months of age. In practice, the logarithm of the spot-forming units (SFU) count was analyzed using Tobit regression (28), a linear model taking into account the lower and upper limits of detection of the assay i.e., left- and right-censoring, respectively. The result was left-censored if the value of the positive control (SEB/CD3) and the values for all five measles pools were all lower than two times the negative control of the plate. The record was right-censored if the median count for at least one of the five pools was above 150 (higher counts would lead to confluence of the ELISpot wells and compromise accurate counting of the number of spots). The outcome measure of interest was the geometric mean count ratio (GMR) in SFU between the MMR and placebo groups. The GMR (with 95% confidence interval) was estimated by exponentiating the corresponding coefficient and its confidence limits resulting from the Tobit model explained above.

All analyses, including the secondary and exploratory analyses explained below, were adjusted for sex assigned at birth and prematurity status (gestational age < 37 weeks) according to the stratified randomization procedure. Further, all analyses were adjusted for the logarithm of the baseline level of the outcome, and all analyses were performed according to the per-protocol principle meaning that the analyses included infants receiving the allocated intervention according to the randomization. An analogous version of the primary analysis but using the SFU measured post-routine MMR at 15 months of age as the outcome was included as a secondary analysis. The secondary analyses also included the post-randomization SFU outcome with effect modification for sex, prematurity status, age at randomization (< 6 months or ≥ 6 months), maternal year of birth as a proxy of immunization status (< 1986; i.e., PIMs, 1986-1987; i.e., unknown status, or > 1987; i.e., PVCs), seroprotection status measured as a dichotomous variable with cutoff at 120 mIU/mL for the PRNT at baseline, and a further categorized version of this variable (levels < 40 mIU/mL, 40-80 mIU/mL, 80-120 mIU/mL, and ≥ 120 mIU/mL). The exploratory analyses included the post-routine MMR SFU outcome assessed for effect modification using the same variables as for the post-intervention analyses mentioned above. Further, for both post-intervention and post-routine MMR SFU outcomes, the GMRs within each of the five pools were analyzed.

For all the analyses mentioned above the analysis set was defined as the infants with an available SFU count at baseline and at post-intervention. However, for the analyses with the post-routine SFU outcome, the results based on the population with available SFU counts at baseline and post-routine were provided in the **Supplementary Material** i.e., omitting the requirement of a post-intervention value.

Data were analyzed using Stata version 18.0.

### Ethics

The protocol was approved by the Capital Region biomedical research ethics committee (H-16041195), the Danish Medicines Agency, and the Danish Data Protection Agency (J.no. 2015-41-4508). The trial was monitored by a steering committee, the Capital Region Good Clinical Practice Unit, and a data safety monitoring board (DSMB). All legal guardians signed informed consent forms prior to participation. The trial was performed in accordance with the principles of the Declaration of Helsinki and reported in accordance with the CONSORT guidelines.

### Role of the funding source

The funder of the study had no role in designing the study, patient recruitment, data collection, analysis, interpretation, writing of manuscripts, decision to submit for publication, or any aspect pertinent to the study.

## Results

### Design and demographics

**Figure 1:**
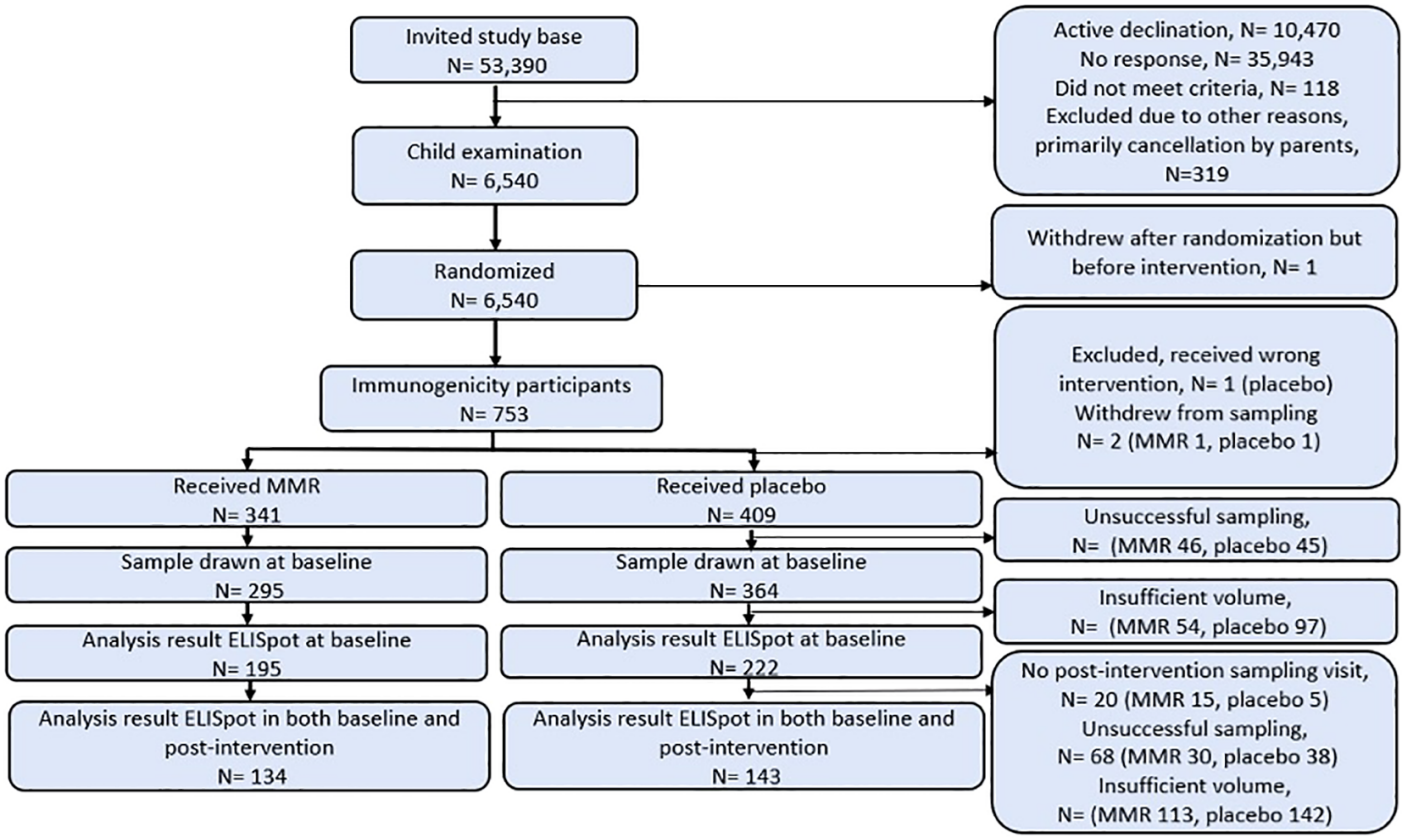
Participant flow chart based on randomization group. For elaboration on samples for post-routine MMR analyses see **Supplementary Figure S1**.

The cellular immunogenicity subpopulation in the Danish MMR trial was defined by participation leading to an ELISpot result before and after intervention. A total of 753 infants were randomized, with 749 receiving their allocated intervention (341 and 409 infants received MMR and placebo, respectively, see **Figure 1**, participant flow chart), and 277 infants donated blood samples voluminous enough at the first and second visit to participate in the main analysis (MMR N= 134, placebo N=143, see **Figure 1**).

Participants’ baseline characteristics were equally distributed across randomization groups. The only difference was detected for prematurity in the population (**Table 1**, demographics based on randomization group, MMR 11.4% vs. placebo 6.7%). Mean time from intervention to post-randomization sampling was 27 (range 19-41) days (**Table 1**). Maternal birth year before and after the introduction of MMR vaccination in Denmark in 1987 was used as a proxy for previous wild-type measles infection (**Table 1**). Based on this assumption, it is likely that a about 50% of the mothers in the trial are PIMs with measles than the self-reported rate of 13 of 264 (or 5%) of the mothers (**Table 1**).

**Table 1:** Baseline characteristics of the cellular immunogenicity subpopulation based on intervention group (early MMR vs. placebo).

	Total N	MMR N (%)	Placebo N (%)
**Baseline characteristics**	277	134 (48.4)	143 (51.6)
**Sex boys**	277	75 (53.2)	76 (56.0)
**Mean infant age months ****^a^**	276	6.5 (6.4-6.5)	6.5 (6.4-6.6)
**Age at randomization < 6 months**	276	13 (9.7)	10 (7.0)
Mean post-int. sampling interval ^b^	276	27.0 (18.5-35.6)	27.7 (20.5-40.5)
Mean post-routine MMR sampling interval ^b^	260	26.0 (3–62)	27.0 (20-78)
**Premature (GA<37 weeks)**	267	15 (11.4)	9 (6.7)
**Number of siblings**	276		
0		70 (52.2)	70 (49.3)
1		40 (29.9)	46 (32.4)
2 or more		24 (17.9)	26 (18.3.)
**Mean maternal age in years** **^c^**	274	32.8 (32.1-33.5)	32.6 (31.9-33.3)
**Mother year of birth**	277		
Before 1986		56 (41.8)	53 (37.0)
Between 1986-1987		20 (14.9)	27 (18.9)
After 1987		58 (43.3)	63 (44.1)
**Household income per year (USD)**	275		
Less than 27000		3 (2.2)	4 (2.8)
Between 27000-54000		19 (14.2)	26 (18.4)
More than 54000		112 (83.6)	111 (78.7)
**Parents living together**	275	128 (95.5)	135 (95.7)
**Mother’s educational level**	276		
≤ High-school education		9 (6.7)	13 (9.2)
Vocational education - bachelor’s degree		60 (44.8)	49 (34.5)
≥ Master’s degree		65 (48.5)	80 (56.3)
**Maternal measles immunization status** **^d^**	264		
Previously infected		4 (3.2)	3 (2.2)
Vaccinated		118 (94.4)	133 (95.7)
Both previously infected and vaccinated		3 (2.4)	3 (2.2)
Not immunized		0 (0.0)	0 (0.0)

The population is defined by participation in the main analysis requiring an ELISpot result at baseline and at post-intervention.

a Mean infant age in months with 95% confidence intervals in parenthesis.

b Mean sampling interval in days since intervention/routine MMR with range in parenthesis.

c Mean mother age in years (95% CI).

d Self-reported immunization status.

### Blood samples

749 mother-infant dyads participated in the immunogenicity studies. Ideally, the mothers donated one blood sample at baseline, whereas the infants donated three blood samples: at baseline (6 months of age), four weeks post-intervention (i.e., at 7 months of age), and four weeks post-routine vaccination (i.e., at 16 months of age). At each time point, the infants were to donate up 14 mL blood, of which 3.5 mL was used to make serum for serological analyses, and the remaining 10.5 mL were used to make PBMCs for cellular immune analysis.

From the infants, however, the actual volumes of blood and the resulting quantities of PBMCs obtained varied considerably. When the full volume of 10.5 mL was obtained, yields per infant were up to 113x10^6^ PBMCs (median 25x10^6^), whereas the PBMC yields per mother were up to 20x10^6^ PBMC (median 11x10^6^). In some cases, the blood samples from the infants were much smaller and/or coagulated and could not be processed successfully.

The serological blood sampling was prioritized and succeeded consistently with enough serum for full serological analysis at all three time points from 563 (or 87%) of the 647 infants participating in the humoral arm of the trial. This supported the high-powered primary serological analysis, which was the primary outcome of the Danish MMR trial (10). In contrast, the cellular blood sampling process, which is of relevance here, was much more challenging. We aimed to obtain three blood samples from each of the infants participating in the cellular arm of the trial. To test five measles peptide pools and two controls in up to four replicates of up to 500,000 cells/well, we ideally needed at least 10 million, preferably 14 million, PBMCs from each sample. The success rate of obtaining enough PBMC’s from the infants was only 56%.

**Figure 2:**
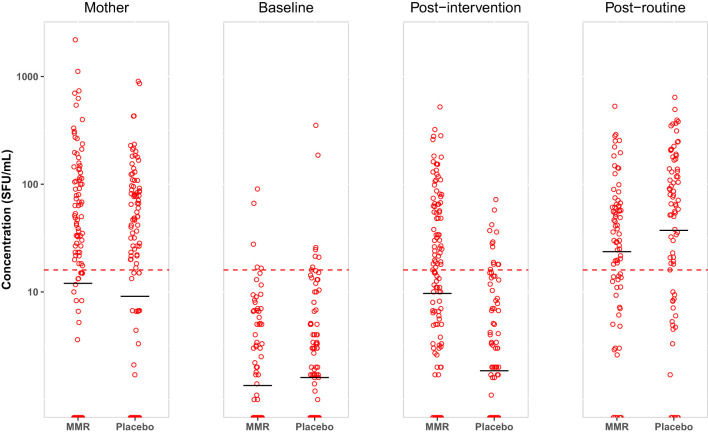
Based on randomization groups (MMR/placebo), measles-specific T cell responses are measured by ELISpot. Data are presented for each time point; mothers at baseline, infants at baseline, post-intervention, and post-routine MMR. The distribution of T cell responses is presented as SFU/10^6^ PBMCs with mean (black horizontal line) on a logarithmic scale. The cellular conversion rate (CCR) is defined for this population to be 16 SFU/10^6^ PBMCs (represented by the dotted red line).

In total, we successfully obtained ELISpot results from 407 infants before intervention, 405 infants after intervention, and 370 infants following routine MMR vaccination (**Figure 2**). 277 of the infants provided PBMCs before and after intervention, and 157 infants provided complete sets of PBMCs representing all three time points (**Supplementary Figure S1**). We obtained blood samples from 735 mothers at baseline and successfully prepared PBMCs from 682 (93%) of these, all of which were successfully tested by ELISpot.

### Cellular immunogenicity of MMR vaccination at 6 months of age

The primary analysis of this report focuses on the cellular immune responses to measles following MMR vaccination administered at 6 months of age. To this end, we used IFN-γ ELISpot analyses to visualize and quantitate the number of measles-specific T cell clones induced by MMR vaccination. The frequencies of measles-specific IFN-γ secreting T cells were calculated and reported as spot-forming units (SFU) per 10^6^ PBMCs. 3-5 weeks after vaccination at 6 months of age with either MMR or placebo, we observed Geometric Mean Counts (GMC) of 11 and 2 SFU/10^6^ PBMCs, respectively, and a GMR of 12.3 (95% CI 6.9-21.9) (**Tables 2**, **3**). This shows stimulation of measles-specific T cells when infants at 6 months of age were vaccinated with MMR.

**Table 2:** Descriptive results of the ELISpot T cell analysis. All descriptive results are based on maximum number of observations.

	MMR				Placebo			
	Mother	Baseline	Post int.	Post routine	Mother	Baseline	Post int.	Post routine
*ELIspot*	N= 313	N= 195	N= 183	N= 163	N= 363	N= 222	N= 224	N= 209
GMC	12 (9-15)	2 (1-2)	11 (8-14)	23 (18-32)	12 (10-16)	2 (1-2)	2 (2-2)	36 (28-47)
*Sex, GMC*	N= (169, 144)	N= (103, 92)	N= (99, 84)	N= (92, 71)	N= (188, 175)	N= (115, 107)	N= (124, 100)	N= (111, 98)
Male	13 (9-18)	1 (1-2)	11 (7-16)	24 (17-35)	11 (7-15)	2 (1-2)	2 (2-3)	41 (29-58)
Female	11 (7-16)	2 (1-2)	10 (7-15)	23 (15-37)	14 (10-20)	2 (1-2)	2 (1-2)	31 (21-47)
*Prematurity, GMC*	N= (25, 284)	N= (21, 172)	N= (19, 161)	N= (14, 146)	N= (11, 340)	N= (10, 202)	N= (12, 203)	N= (8, 195)
GA <37	17 (6-42)	1 (1-1)	7 (2-19)	14 (3-61)	10 (2-64)	2 (1-5)	1 (0-4)	18 (4-82)
GA ≥37	11 (9-15)	2 (1-2)	12 (9-16)	25 (19-34)	12 (9-16)	2 (1-2)	2 (2-3)	37 (28-48)
*Age at intervention, GMC*	N= (44, 269)	N= (24, 171)	N= (19, 164)	N= (17, 146)	N= (37, 326)	N= (17, 205)	N= (20, 204)	N= (14, 195)
< 6 months	10 (5-19)	3 (1-5)	11 (4-32)	20 (9-44)	20 (9-45)	2 (1-3)	2 (1-6)	52 (21-126)
≥ 6 months	12 (9-16)	4 (2-5)	11 (8-14)	24 (18-33)	12 (9-15)	2 (1-2)	2 (2-2)	35 (27-47)
*Mother year of birth, GMC*	N= (136, 47, 130)	N= (77, 29, 89)	N= (84, 27, 72)	N= (66, 33, 64)	N= (139, 65, 159)	N= (87, 39, 96)	N= (83, 40, 101)	N= (79, 42, 88)
Before 1986	12 (8-18)	2 (1-2)	8 (5-12)	20 (12-33)	12 (8-17)	2 (1-3)	2 (1-2)	40 (27-61)
1986-1987	13 (7-25)	2 (1-2)	17 (9-35)	27 (14-51)	14 (8-26)	2 (1-3)	3 (1-5)	30 (17-52)
After 1987	11 (8-17)	2 (1-2)	12 (8-19)	27 (18-40)	12 (8-18)	2 (1-2)	2 (1-3)	36 (24-56)
*PRNT baseline, GMC*	N= (231, 39)	N= (168, 26)	N= (138, 27)	N= (128, 17)	N= (275, 41)	N= (191, 26)	N= (172, 32)	N= (156, 26)
<120 mIU/mL	12 (9-17)	2 (1-2)	11 (8-15)	22 (16-31)	12 (9-16)	2 (1-2)	2 (2-3)	34 (25-47)
≥120 mIU/mL	9 (4-20)	2 (1-3)	6 (3-12)	24 (11-52)	13 (6-29)	2 (1-3)	2 (1-4)	55 (32-95)
*PRNT baseline categorized, GMC^£^*	N= (168, 45, 18, 39)	N= (121, 33, 14, 26)	N= (97, 30, 11, 27)	N= (91, 27, 10, 17)	N= (180, 68, 27, 41)	N= (126, 44, 21, 26)	N= (113, 41, 18, 32)	N= (104, 36, 16, 26)
0-40 mIU/mL	11 (8-16)	1 (1-2)	13 (9-20)	22 (15-31)	11 (8-16)	2 (2-2)	2 (2-3)	40 (27-58)
40-80 mIU/mL	15 (7-33)	2 (1-3)	8 (4-16)	26 (13-54)	11 (6-20)	2 (1-3)	2 (1-3)	23 (11-48)
80-120 mIU/mL	19 (6-66)	1 (0-2)	5 (2-13)	18 (2-162)	30 (13-70)	1 (1-3)	2 (1-4)	32 (13-81)
>120 mIU/mL	9 (4-20)	2 (1-3)	6 (3-12)	24 (11-52)	13 (6-29)	2 (1-3)	2 (1-4)	55 (32-95)

Post int., Post-intervention sample 3-5 weeks after MMR/placebo at 5-7 months of age. Post routine, Post routine MMR sample 3-5 weeks after MMR1 at 15 months of age. GMC, geometric mean count in SFU/10^6^ PBMCs (95% CI). N= (XX, YY) refers to XX individuals in first mentioned subgroup (e.g. Male) and YY individuals in other subgroups (e.g. Female). ^£^ PRNT Baseline: Concentration of measles-neutralizing antibodies in infant baseline samples. Baseline PRNT result is missing (N) for MMR: 1, 18, 18, 27 and placebo: 5, 20, 27, 29. Descriptive ELISpot results with arithmetic means and ranges are given in **Supplementary Table S3**.

**Table 3:** Analytical results of measles-specific T cells based on ELISpot measurements.

	Post intervention (MMR/placebo)		Post routine MMR (MMR/placebo)	
	N^¤^	GMR	N	GMR
Measles specific T cells				
GMR	134;143	12.3 (6.9-21.9)	79;78	0.6 (0.3-0.9)
*Effect modification*				
Sex				
Male	75;76	9.8 (4.6-20.7)	48;42	0.4 (0.2-0.9)
Female	59;67	16.4 (6.9-38.9)	31;36	0.8 (0.4-1.7)
*Prematurity*				
GA <37	15;9	31.2 (2.6-371.2)	8;5	2.7 (0.4-17.5)
GA ≥37	117;126	11.6 (6.4-21.0)	69;69	0.5 (0.3-0.8)
*Age at intervention*				
< 6 months	13;10	40.3 (5.4-302.2)	6;6	1.3 (0.2-8.3)
≥ 6 months	121;133	22.7 (4.2-123.3)	73;72	0.6 (0.2-2.3)
*Mother year of birth*				
Before 1986	56;53	4.9 (2.2-11.0)	32;31	0.4 (0.2-0.9)
1986-1987	20;27	17.7 (6.0-51.6)	16;16	0.6 (0.2-1.5)
After 1987	58;63	15.0 (6.8-33.3)	31;31	0.6 (0.3-1.3)
*Baseline PRNT*				
< 120 mIU/mL	111;122	12.1 (6.5-22.6)	68;63	0.6 (0.3-1.0)
≥ 120 mIU/mL^§^	22;17	4.6 (1.6-12.7)	10;11	0.6 (0.2-1.7)
*Baseline PRNT*				
< 40 mIU/mL	76;81	12.5 (6.1-25.8)	45;43	0.5 (0.3-1.0)
40-<80 mIU/mL	25;29	6.7 (2.6-17.4)	16;14	0.6 (0.2-1.4)
80-<120 mIU/mL	10;12	4.3 (1.0-17.9)	7;6	0.4 (0.1-1.7)
≥ 120 mIU/mL^§^	22;17	3.8 (1.3-10.8)	10;11	0.5 (0.2-1.6)

The results are based on the main cellular immunogencity population; i.e., infants having a T cell measurement at baseline and at the post-intervention time point.

For post routine MMR evaluation, only infants with non-missing samples at all three time points are included.

GMR is the geometric mean count ratio between early MMR and placebo groups. Post-intervention: Sample collected 3-5 weeks after MMR/placebo at 5-7 months of age. Post-routine MMR: Sample collected 3-5 weeks after MMR at 15 months of age.

^¤^ XX; YY refers to X individuals in MMR group and Y individuals in placebo group.

^§^ Note that the GMR corresponding to the ≥ 120 mIU/mL baseline PRNT group may differ in the two categorizations of the variable due to small differences in the underlying models.

### Effect modification of the cellular immunogenicity of early MMR

No significant effect modification was observed. However, low levels of maternal measles-neutralizing antibodies tended to be associated with a higher T cell response after early MMR compared to placebo (**Table 3**). Similar tendencies for a higher T cell response were found for proxies for low levels of maternal antibodies such as prematurity or maternal year of birth being after 1986. Sex and infant age at intervention did not affect the T cell responses after early MMR (**Table 3**). No significant differences were seen between the different measles proteome pools (**Supplementary Tables S4, S5**).

### T cell response after repeated MMR

In accordance with the Danish NIP, all participating infants received a routine MMR vaccination at 15 months of age. For infants in the placebo group, this was the primary MMR vaccination; for those in the MMR group, it was the second MMR vaccination. In a short-term follow-up, we measured the measles-specific immune responses in these infants 3-5 weeks after their routine vaccination at 15 months of age. As previously reported, the serological responses in the MMR and placebo groups were 1804 and 1174 mIU/ml, respectively, yielding a statistically significant serological GMR of 1.5 (95% CI 1.3–1.9) (10) demonstrating that the serological immune response as measured by PRNT is significantly higher after a secondary vaccination at 15 months of age than after a primary vaccination at the same age. In stark contrast to this, we here observed the opposite pattern: the GMCs of the cellular responses in the MMR and placebo groups were 23 vs. 36 SFU/10^6^ PBMCs (**Table 2**), respectively, resulting in a cellular GMR of 0.6 (95% CI 0.3-0.9) (**Table 3**). Thus, the relative cellular immune response after routine vaccination at 15 months of age as measured by IFN-γ ELISpot analyses was significantly lower in the MMR intervention group compared to the placebo intervention group reflecting the ability of vaccine-induced pre-existing immunity to effectively contain a new vaccine-induced measles challenge. No significant effect modifications were observed on post-routine MMR responses (**Table 3**, **Supplementary Tables S2, S6, S7**).

### T cell and serological responses

The co-primary immunogenicity outcome of the Danish MMR trial was to evaluate the specific humoral immune responses elicited by MMR vaccination at 6 months of age; findings that have been detailed in a prior publication (10). Here, these findings are recapitulated for context and to enable comparisons with the cellular immune responses. The primary serological assessment was done using a standardized plaque reduction neutralization test (PRNT) to measure the geometric mean concentrations (GMC) of measles neutralizing antibodies. One month after vaccination at 6 months of age with either MMR or placebo, we observed GMCs of 120 and 25 mIU/ml, respectively, corresponding to a significant geometric mean ratio (GMR) of 4.3 (95% CI: 3.4–5.3) (10). This was the primary immunogenicity outcome of the trial showing that MMR vaccination at 6 months of age raises a significant humoral immune response. However, not every infant vaccinated with MMR at 6 months developed a measurable and/or protective serological response against measles. The seroconversion and seroprotection rates, SCR and SPR were 49% and 45%, respectively (10). Although this compared favorably with the placebo group, where the SCR and SPRs were 7% and 11%, respectively, the immunogenicity of MMR vaccination at 6 months of age was less than expected, probably due to the persistence of maternally derived measles-neutralizing antibodies at 6 months of age. Encouragingly, a subsequent MMR vaccination at 15 months of age increased the measles-specific SCR and SPR to 98%, respectively, slightly surpassing the SCR and SPR of 95% and 96%, respectively, observed after the primary routine MMR vaccination at 15 months of age (10). Thus, the reduced SCR and SPR after MMR vaccination at 6 months could be boosted and may not necessarily reflect a permanent reduction but instead rather be a temporary effect of maternal antibodies.

A critical measure of the efficacy of early MMR vaccination is the resulting totality of specific immune responses in a large proportion of the receiving infants. The SCR after MMR vaccination at 6 months of age was 49% (10). Notions of a comparable T cell or cellular conversion rate (CCR, see Methods section for a definition), much less of a cellular protection rate, do not appear to be in general use (29–31). Here we observed a CCR of 45% and 9% after intervention (MMR or placebo, respectively) at 6 months of age, which compared well to the observed SCR of 45% (**Table 4**). Interestingly, however, there was not complete agreement between the serological and cellular conversion rates since approximately 40% of the infants displayed discordant results, equally distributed as positive SCR, but negative CCR, or vice versa (**Table 4**). Comparing the SCR and CCR following routine vaccination at 15 months of age, they were 97% and 68%, respectively, in the MMR group and 96% and 75%, respectively, in the placebo group, suggesting that almost all infants achieved seroconversion after the routine MMR vaccination at 15 months of age irrespective of whether they had been given an early MMR vaccination at 6 months of age, or not. In contrast, the cellular conversion rate was slightly reduced in the MMR group, probably reflecting that pre-existing immunity effectively inhibited a new vaccine-induced measles challenge and reduced the cellular response against the secondary routine vaccination. Based on this approach, we observed the totality of serological and cellular measles-specific immune conversion rates to be 64% and 99% after early MMR and after routine MMR, respectively. In parallel, the placebo group showed immune conversion rates of 15% and 99% after placebo intervention and after routine MMR, respectively. This indicates that nearly two-thirds of the early vaccinated infants mounted a specific immune response, and early vaccination did not reduce the proportion of infants mounting a response after routine vaccination. For comparison, the mothers had SPR at 91% and 83%.

**Table 4:** Results related to conversion and protection rates.

	MMR				Placebo			
	Mother	Baseline	Post int.	Post routine	Mother	Baseline	Post int.	Post routine
*Seroprotection rate (SPR%)*	N= 133	N= 133	N= 129	N= 114	N= 139	N= 139	N= 137	N= 118
	83%	17%	45%	98%	91%	12%	11%	96%
*Seroconversion rate (SCR%)*	-	-	N= 129	N= 114	-	-	N= 135	N= 121
	–	–	49%	87%	–	–	7%	96%
*T cell conversion rate (CCR%)*	N= 129	N= 134	N= 134	N= 79	N= 134	N= 143	N= 143	N= 78
	52%	4%	45%	66%	51%	6%	9%	74%
*Immune conversion rate (ICR%)*	-	-	N= 129	N= 72	-	-	N= 135	N= 71
*- Both T cells and serology*		–	29%	61%		–	1%	77%
*- Only seroconversion*			19%	32%			5%	21%
*- Only T cell conversion*			16%	6%			8%	0%
***- Any conversion***			**64%**	**99%**			**15%**	**99%**
*- No conversion*	–		36%	1%	–		85%	1%

Conversion rates can only be calculated for individuals with pre- and post- immunization samples and lab. analyses.

The seroconversion rate (SCR) is the proportion of infants achieving either a four-fold increase in the concentration of antibodies from pre-intervention (or routine MMR) to post-intervention, or changing status from unprotected to protected according to the protective cutoff at 120 mIU/mL. Accordingly, the seroprotection rate (SPR) is calculated as the proportion of individuals with a level above 120 mIU/mL.

The T cell conversion rate (CCR) is the proportion of individuals having a measurable level of measles-specific T cells, i.e., above the 95^th^ percentile for the total baseline samples at 16 SFU/10^6^ PBMCs.

Combining SCR and CCR, the immune conversion rate (ICR) reports the proportion of infants achieving any immune conversion (seroconversion and/or cellular conversion) following intervention MMR or routine MMR (shown in bold as "Any conversion").

The calculations are based on the maximum number of observations within the main population, i.e., mother-infant dyads participating in the main analysis.

## Discussion

The Danish MMR trial was a double-blind, placebo-controlled, randomized clinical trial conducted from April 2019 to January 2022. It aimed to explore the feasibility of moving the first MMR vaccination forward to 6 months of age thereby closing the time gap that has emerged between the waning of protective maternal antibodies, which happens earlier in infants born by previously vaccinated mothers (PVMs) than by previously infected mothers (PIMs) (7), and the induction of active immunity, which does not occur until after the first MCV vaccination. In Denmark, the first MMR is currently administered at 15 months of age; advancing this to 6 months of age could potentially reduce the vulnerable time gap by up to 9 months. We have previously reported the primary outcome from the Danish MMR trial, which showed that administering the first MMR vaccine dose at 6 months of age is safe and not associated with higher rates of reactogenicity than placebo. It induced a significant humoral immune response albeit with seroconversion and seroprotection rates of both 47%. It did not negatively impact short-term responses to a subsequent routine MMR vaccination (10). In the present report, we focus on the secondary outcome of the Danish MMR trial, specifically the generation of measles-specific IFN-γ secreting T cells, as measured by ELISpot assays. To the best of our knowledge this is the first randomly controlled trial that has examined cellular immune responses resulting from early measles vaccination.

### The cellular immunogenicity of early MMR vaccination

To assess the immunogenicity of an early MMR vaccination, both the magnitude and the prevalence of the resulting immune response must be considered. Here, we demonstrate that MMR vaccination at 6 months of age, compared to placebo vaccination, leads to a significant increase in the magnitude of measles-specific T cell responses (note, whereas the serological response to early MMR vaccination is reduced in infant’s younger vs older than 6 months of age, the cellular response is not). An equally important consideration is the prevalence of inducing cellular immune responses in the early vaccinated infants. Whereas the prevalence of a humoral immune responses is conventionally expressed as a seroconversion rate (SCR), there are no similar concepts in common use to describe the prevalence of cellular immune responses. We would like to suggest that the prevalence of a cellular immune responses can be expressed in a straightforward manner as the proportion of vaccinees who have achieved a defined cellular response level. This has recently been referred to by others as a cellular conversion rate (CCR) (29–31). In this study, we used ELISpot to count T cells responding to an overlapping peptide library representing the entire measles proteome, measured as spot-forming units (SFU). We defined an infant in the MMR intervention group with a measles-specific T cell response greater than the 95^th^ percentile of the placebo group as being cellularly converted. By this definition, a cellular conversion rate, CCR, of 45% was calculated in the MMR intervention group. In total, 29% of the infants converted both serologically and cellularly, while 19% converted solely serologically and 16% solely cellularly. In summary, 64% of these infants mounted a serological and/or a cellular immune response following an early MMR vaccination. The observation that some infants converted solely serologically or cellularly is consistent with prior findings by others. Thus, using Yellow Fever vaccination as a model system, Pulendran and Ahmed have shown that immune responses can be individually skewed, with some individuals mounting predominantly serological responses, while others exhibit a stronger cellular or innate immune response (32, 33).

### Cellular protection rate

Rather than addressing serological and/or cellular conversion rates, it is more clinically relevant to address the ability of a vaccine to induce a protective immune response. For serological responses, the protective level of measles-specific neutralizing antibodies is conventionally considered to be 120 mIU/mL. This biochemical threshold is routinely used to determine the proportion of measles vaccinees that successfully obtain protective levels of antibodies and is expressed as the “seroprotection rate” (SPR), a convenient measure of vaccine efficacy. A corresponding concept of a “cellular protection rate” (CPR) is not currently in use, probably because it is a very complex and challenging parameter to assess and due to a limited understanding and appreciation of the impact of cellular protection mechanisms. Further complicating this issue, the randomized controlled vaccine trials needed to assess a CPR are inherently complicated by the extreme HLA-mediated diversification of human cellular immune responses. Using a recombinant mouse model, we recently demonstrated a protective role of T cell responses in Yellow Fever vaccination, but we had to resort to inbred animals with B cell deficiencies before the protective effect of T cells could be observed (34). Only recently, using defined antigens to induce influenza-virus-specific CD8+ T cell responses in inbreed animal models, it has been possible to demonstrate that CD8+ T resident memory cells can inhibit virus transmission causing near-sterile immunity (35). In summary, the concept of a cellular protection rate is a highly desirable, albeit currently elusive, goal.

### Routine vaccination at 15 months

In terms of response magnitude, we observed a significantly diminished cellular immune response following a secondary catch-up (or routine) MMR vaccination at 15 months in the MMR intervention group compared to the placebo group. We and others have previously observed a similar phenomenon of reduced cellular responses to booster vaccinations with attenuated Yellow Fever vaccines (24, 36). It has been shown that this can be caused by pre-existing immunity reducing the take of subsequent vaccines (36). This contrasts with our serological findings where a secondary vaccination significantly enhanced the magnitude of the humoral immune response (10). Viewing secondary live-virus vaccinations as a proxy for a challenge experiment, we interpret the findings of reduced secondary vaccination cellular responses to measles as an indication that the primary MMR vaccination at 6 months of age has successfully managed to establish a protective immune response. Thus, we speculate that diminished cellular secondary immunity may be a sensitive correlate of protection. In this context, we propose that the measles component of the MMR vaccine, when administered to infants at 6 months of age, is immunogenic and raises both a humoral and cellular immune response.

In terms of cellular conversion rates in the present trial, in the MMR intervention group, the secondary MMR vaccination at 15 months of age increased the SCR and CCR from 49% and 45% after the early primary MMR vaccination to 87% and 66%, respectively, after the secondary MMR vaccination. By comparison, in the placebo intervention group, the routine MMR vaccination at 15 months of age gave a SCR and CCR of 96% and 74%, respectively. Thus, the CCR is reduced after the MMR vaccination at 15 months of age in the early MMR vs. placebo groups mirroring the effect of early vaccination on the magnitude of the response. At first glance this shows that a secondary MMR vaccination at 15 months of age leads to a diminished cellular immune response compared to a primary vaccination at the same age, however, this does not necessarily mean that the infants in the MMR group have less immunity against measles than the infants in the placebo group after routine MMR. Instead, we propose that the relatively reduced cellular immune response to the routine vaccination in the MMR group is a proxy for the immune protection afforded by the primary vaccination. Rather than seeing this result as a relative failure of the secondary vaccination at 15 months of age, one could see this as a relative success of the primary vaccination at 6 months of age. In addition, it is reassuring that the relatively reduced MMR vaccine response at 6 months of age is only a temporary effect, and that all infants generate strong serological and cellular immune responses after routine MMR vaccination at 15 months of age irrespective of early MMR at least when being assessed short-term. It remains to evaluated whether long-term immune responses will persist on par between the two groups.

### Prior research

Gans and coworkers studied measles-specific cellular immune responses in a cohort of infants that had received an early MCV prior to a routine two-dose MMR vaccination schedule (administered at 12 months and 5 years of age). They used a ^3^H-thymidine incorporation assay to measure T-cell proliferation and reported the results as a stimulation index. They found that the timing of the primary vaccination (whether at 6, 9, or 12 months of age) did not significantly impact the magnitude and prevalence of measles-specific T cell responses detected 12 weeks after the primary vaccination (37). When infants who received their primary measles vaccination at 6 or 9 months were subsequently revaccinated with MMR at 12 months of age, their measles response was significantly higher than 12-month-old infants who had only been given a primary MMR vaccination (38). Long-term follow-up studies at 5–10 years of age revealed that measles-specific T-cell responses were comparable irrespective of the timing of the primary vaccination (39). Our finding that the post-routine response in the MMR group is significantly lower than in the placebo group appear to contradict the results of Gans and co-workers. However, it is important to note that there are significant differences in the experimental design and read-outs between their studies and ours, including differences in the timing of revaccination (12 vs. 15 months) and the methods used to measure the T cell response (T cell proliferation vs. ELISpot; stimulation index vs. response difference).

### Measles is still an important infectious disease

Measles virus remains a serious threat to human health. With a high basic reproductive number (9–18), this airborne virus is one of the most contagious microorganisms known (40). Before the introduction of measles vaccination, the disease was endemic in exposed populations with recurrent epidemic outbreaks every 2-5 years (2, 41). Most individuals contracted the disease at an early age and became immune and protected against reinfection for life (42). The widespread immunity that resulted from these infection cycles may have masked measles as being a harmless infection of childhood. In reality, in unprotected individuals, particularly in the very young and in the elderly, measles causes severe and potentially fatal complications (43–45). The serious nature of measles infections has most dramatically been seen when measles has been introduced into unexposed populations causing “first-contact epidemics”; something that has happened historically, e.g. when measles was introduced to the Americas, where it caused high double-digit death rates in the hitherto unexposed indigenous populations (44), or when small, remotely located and unexposed populations contracted measles sporadically from infected visitors (43).

### Vaccines protects against measles

Before the introduction of vaccines, measles worldwide caused >30 million cases and >2 million deaths per year (46); after the introduction of vaccines, the worldwide incidence had dropped to 9 million cases and 136000 deaths in 2022 (actually, an increase over previous years) (3). Thus, to this day, measles remains a serious infection with case fatality ratios in children from 0.01% in developed countries to 5% in developing countries. A particularly worrisome aspect of measles infection has recently come to light: the virus infects immune memory cells thereby possibly erasing the accumulated immune memory of past infections of the infected individual, causing a vulnerable state of immune suppression and “immune amnesia” (47–49), something that is particularly troublesome in the elderly, who heavily depend on immune memory cells.

Measles is a vaccine-preventable disease. Safe and effective live-attenuated vaccines exist and are widely used, either alone or in combination with other vaccines. WHO recommends a two-dose vaccination regime where the first dose is given at 9 months of age in endemic areas and at 12-15 months in non-endemic areas (5) The efficacy of the first dose may be inhibited by maternal anti-measles antibodies leading to vaccine failure in as much as 10-15% of children vaccinated at 9 months of age (5). Therefore, WHO recommends that a second “catch-up” dose be given at a later time point to ensure high coverage of children who are actively immunized. Given the very contagious nature of measles infection, a 95% coverage rate is needed globally to eliminate the measles virus, however, the global coverage is far from reaching that goal (50). This is regrettable since the biological conditions for complete eradication of measles virus exists (51, 52). In 2010, the Strategic Advisory Group of Experts (SAGE) concluded “that measles can and should be eradicated” (53). Albeit working toward this goal, it has not yet been formally stated by the WHO (54). In contrast, during and after the recent COVID-19 pandemic, there has been as worldwide resurgence of measles due to decreased vaccine coverage rates (55–57). The need for high measles vaccine coverage, optimal vaccination timing and protection of increasingly vulnerable infants serves as a compelling rationale for this study.

### Limitations

The serological responses were the main outcomes of the Danish MMR trial and had priority over measuring the cellular responses, which were secondary outcomes. This prioritization came with a trade-off. Whereas the selected sampling time of four weeks post-vaccination is optimal for serological analysis, it is not an optimal sampling time for cellular immunology analysis. Here, a more optimal timing would have been 14 days after vaccination. Another drawback is that a significant number of infant samples failed to provide the amount of PBMC needed for ELISpot analysis thus reducing the statistical power of the cellular outcome.

The effectiveness of early MMR against clinical disease could not be evaluated due to the scarcity in measles cases in Denmark.

### Strengths

The randomized controlled trial design is a major strength of the present study, diminishing the risk of bias. Further, the relatively large collection of longitudinal blood samples in the infants, with an additional blood sample from their mothers opened for detailed analyses regarding the role of maternal antibodies (see supplementary discussion on the effect of maternal antibodies), and the effect of the early intervention with an MMR at 6 months of age on the subsequent routine MMR-vaccine at 15 months of age, which was administered to all trial participants.

## Conclusion

This analysis of measles-specific cellular immune responses following MMR vaccination at 6 months of age aligns well with the previously reported serological responses (10) supporting our overall conclusion that the measles component of the MMR vaccine is immunogenic in infants vaccinated at 6 months of age, where it elicits significant humoral and cellular immune responses. In total, 64% of the infants vaccinated with the MMR vaccine at 6 months of age induced a significant post-intervention, measles-specific humoral and/or cellular immune response. When subsequently given, the routine MMR vaccination at 15 months of age resulted in 98% and 99% of the infants showed significant post-routine humoral and/or cellular immune responses, whether they had received a placebo or MMR vaccination at 6 months of age, respectively, indicating that an early 6-month intervention did not compromise the short-term immune effects of the 15-month routine vaccination.

## Data Availability

The data will be available upon reasonable request to “Rigsarkivet”. Requests to access the datasets should be directed to lone.graff.stensballe@regionh.dk.
