# Supplementary material for: Measles-mumps-rubella-vaccination at 6 months of age induces measles-specific T cell responses: a randomized controlled trial
Source: Front Immunol. 2025 Mar 17;16:1546253. doi: 10.3389/fimmu.2025.1546253 (PMC11955646; doi:10.3389/fimmu.2025.1546253)
Supplement: Supplementary file 1 [file DataSheet1.docx]

# Supplementary material

Content

[Supplementary material 1](#_Toc185249422)

[Participant flow chart including all sample types 2](#_Toc185249423)

[Positive controls in ELISpot for IFN-g measles-specific T cells 3](#_Toc185249424)

[Censoring table for analytical results (supporting Table 3 in main text) 4](#_Toc185249425)

[Descriptive ELISpot results with arithmetic means and ranges 5](#_Toc185249426)

[Analytical results for ELISpot measles-proteome pools A-E 6](#_Toc185249427)

[Full population, post-routine MMR results (all infants with a baseline and a post-routine MMR T cell analysis result, irrespective of whether a post-intervention analysis result was successfully obtained, or not) 7](#_Toc185249428)

[Censoring table for full population post-routine MMR analytical results (supporting supplementary table 6) 8](#_Toc185249429)

[Supplementary discussion on the effect of maternal antibodies 9](#_Toc185249430)

[References 10](#_Toc185249431)

## Participant flow chart including all sample types


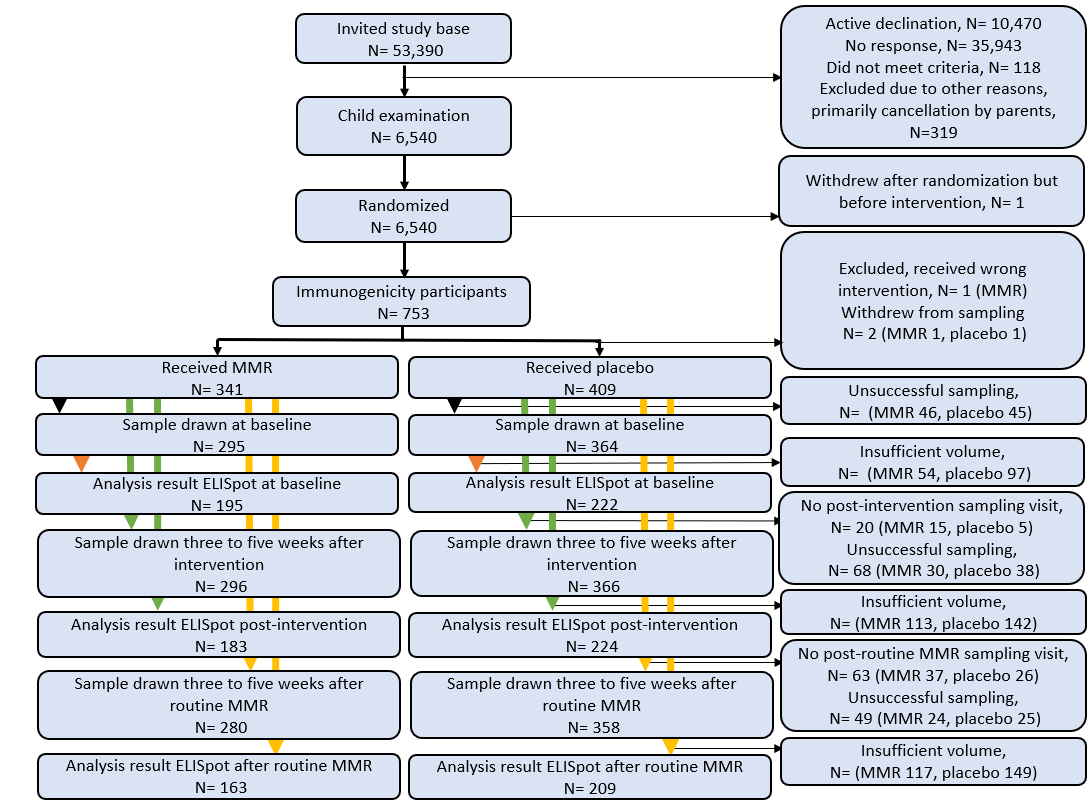


**Supplementary Figure 1**: Participant flow chart for all sample types and T-cell analyses. The orange arrow reflects the infant sampling at baseline and the success rate for processing these samples in the lab. Correspondingly, the green arrows reflect the infant sampling at post-intervention and the success rate for processing these samples in the lab, and for the yellow arrows, these reflect the infant sampling at post-routine MMR and the success rate for processing these samples in the lab.

## Positive controls in ELISpot for IFN-g measles-specific T cells

|  | SEB | | | | Anti-CD3 | | | |
| --- | --- | --- | --- | --- | --- | --- | --- | --- |
| Donor | Total# | >0 | % resp | Median | Total# | >0 | % resp | Median |
| 6 mth | 211 | 149 | 71% | 33 | 206 | 119 | 58% | 110 |
| 7 mth | 152 | 119 | 78% | 68 | 255 | 162 | 64% | 42 |
| 16 mth | 3 | 3 | 100% | 417 | 369 | 308 | 64% | 278 |
| Adults | 217 | 207 | 95% | 1033 | 465 | 409 | 88% | 1617 |

**Supplementary Table 1:** Positive controls for the ELISpot analysis. We have in the past used polyclonal T cell stimulation with Staphylococcal Enterotoxin B (SEB) or anti-CD3 antibodies interchangeably as positive controls to validate the performance of ELISpot assays in adult PBMC samples [1]. Here we observed a reduced capacity of SEB and anti-CD3 to induce polyclonal stimulation of infant T cells, compromising the utility of these as positive controls in ELISpot analysis of infant T cell responses. Thus, the criteria for positive validation of ELISpot analysis were modified: if a given ELISpot setup encompassed at least one donor with a validating positive control, the entire set-up was considered validated.

## Censoring table for analytical results (supporting Table 3 in main text)

|  | Post intervention | | | | | |  | Post routine MMR | | | | |
| --- | --- | --- | --- | --- | --- | --- | --- | --- | --- | --- | --- | --- |
|  | < lower | | > upper | | | |  | < lower | | > upper | | |
|  | MMR | Placebo | MMR | | Placebo | |  | MMR | Placebo | | MMR | Placebo |
| *Analysis* |  |  |  |  | | |  |  |  | |  |  |
| Primary | 39 | 104 | 2 | | | 0 |  | 13 | 11 | | 1 | 2 |
| Sex, male | 23 | 51 | 0 | | | 0 |  | 9 | 7 | | 0 | 1 |
| Sex, female | 16 | 53 | 2 | | | 0 |  | 4 | 4 | | 1 | 1 |
| GA <37 | 4 | 8 | 0 | | | 0 |  | 2 | 2 | | 0 | 0 |
| GA ≥37 | 33 | 88 | 2 | | | 0 |  | 10 | 7 | | 1 | 2 |
| < 6 months | 4 | 8 | 1 | | | 0 |  | 1 | 0 | | 0 | 0 |
| ≥ 6 months | 35 | 96 | 1 | | | 0 |  | 12 | 11 | | 1 | 2 |
| MYOB <1986 | 24 | 41 | 0 | | | 0 |  | 8 | 4 | | 0 | 0 |
| MYOB 1986-1987 | 5 | 19 | 0 | | | 0 |  | 2 | 3 | | 0 | 0 |
| MYOB >1987 | 10 | 44 | 0 | | | 0 |  | 3 | 4 | | 0 | 0 |
| PRNT dic: < 120 mIU/mL | 28 | 86 | 2 | | | 0 |  | 11 | 10 | | 1 | 1 |
| PRNT dic: ≥ 120 mIU/mL | 11 | 15 | 0 | | | 2 |  | 1 | 0 | | 0 | 1 |
| PRNT cat: < 40 mIU/mL | 19 | 53 | 2 | | | 0 |  | 8 | 5 | | 1 | 1 |
| PRNT cat: 40-80 mIU/mL | 6 | 24 | 0 | | | 0 |  | 2 | 5 | | 0 | 0 |
| PRNT cat: 80- 120 mIU/mL | 3 | 9 | 0 | | | 0 |  | 2 | 0 | | 0 | 0 |
| PRNT cat: ≥ 120 mIU/mL | 11 | 15 | 0 | | | 0 |  | 1 | 0 | | 0 | 1 |

**Supplementary Table 2:** The analytical results are calculated using a Tobit regression model (see main text for explanation). Here, the number of sample results that are either left-censored (having a result lower than the lowest limit of detection) or right-censored (having a result higher than the highest limit of detection) are presented for each of the analyses. This table corresponds to the results presented in Table 3 in the main text. For the ELISpot, the lower limit of detection is defined as 2 times the negative control well (left censoring). Upper limit of detection defined is defined as more than 150 spots in at least one of the wells (right censoring). MYOB = Mother year of birth.

## Descriptive ELISpot results with arithmetic means and ranges

|  | **MMR** | | | |  | **Placebo** | | | |
| --- | --- | --- | --- | --- | --- | --- | --- | --- | --- |
|  | Mother | Baseline | Post int. | Post routine |  | Mother | Baseline | Post int. | Post routine |
| *ELIspot* | N= 313 | N= 195 | N= 183 | N= 163 |  | N= 363 | N= 222 | N= 224 | N= 209 |
| Mean (range) | 87  (0-2192) | 3  (0-90) | 40  (0-522) | 71  (0-750) |  | 81  (0-1557) | 6  (0-352) | 7  (0-183) | 105  (0-919) |
| *Sex, mean (range)* | N= (169,  144) | N= (103,  92) | N= (99,  84) | N= (92,  71) |  | N= (188,  175) | N= (115,  107) | N= (124,  100) | N= (111,  98) |
| Male | 88  (0-1345) | 3  (0-67) | 40  (0-278) | 69  (0-750) |  | 77  (0-1043) | 8  (0-352) | 8  (0-183) | 112  (0-767) |
| Female | 87  (0-2192) | 4  (0-90) | 41  (0-522) | 74  (0-613) |  | 85  (0-1557) | 5  (0-154) | 5  (0-101) | 97  (0-919) |
| *Prematurity, mean (range)* | N= (25,  284) | N= (21,  172) | N= (19,  161) | N= (14,  146) |  | N= (11,  340) | N= (10,  202) | N= (12,  203) | N= (8,  195) |
| GA <37 | 85  (0-690) | 1  (0-7) | 23  (0-110) | 69  (0-255) |  | 53  (0-217) | 3  (0-21) | 4  (0-32) | 47  (0-157) |
| GA ≥37 | 88  (0-2192) | 4  (0-90) | 43  (0-522) | 72  (0-750) |  | 82  (0-1557) | 7  (0-352) | 7  (0-183) | 106  (0-919) |
| *Age at intervention, mean (range)* | N= (44,  269) | N= (24,  171) | N= (19,  164) | N= (17,  146) |  | N= (37,  326) | N= (17,  205) | N= (20,  204) | N= (14,  195) |
| < 6 months | 87  (0-1345) | 6  (0-28) | 48  (0-522) | 44  (0-255) |  | 91  (0-563) | 3  (0-15) | 9  (0-101) | 91  (0-190) |
| ≥ 6 months | 88  (0-2192) | 3  (0-90) | 40  (0-322) | 74  (0-750) |  | 80  (0-1557) | 7  (0-352) | 6  (0-183) | 106  (0-919) |
| *Mother year of birth, mean (range)* | N= (136,  47,130) | N= (77,  29,89) | N= (84,  27,72) | N= (66,  33,64) |  | N= (139,  65,159) | N= (87,  39,96) | N= (83,  40,101) | N= (79,  42,88) |
| Before 1986 | 98  (0-1345) | 4  (0-90) | 34  (0-282) | 71  (0-750) |  | 86  (0-1557) | 8  (0-352) | 3  (0-29) | 119  (0-919) |
| 1986-1987 | 98  (0-2192) | 2  (0-9) | 49  (0-278) | 71  (0-613) |  | 79  (0-808) | 6  (0-154) | 8  (0-42) | 78  (0-348) |
| After 1987 | 71  (0-736) | 3  (0-67) | 45  (0-522) | 71  (0-613) |  | 78  (0-1043) | 5  (0-186) | 9  (0-183) | 105  (0-640) |
| *PRNT baseline, mean (range)* | N= (231,  39) | N= (168,  26) | N= (138,  27) | N= (128,  17) |  | N= (275,  41) | N= (191,  26) | N= (172,  32) | N= (156,  26) |
| <120 mIU/mL | 93  (0-2192) | 3  (0-90) | 44  (0-522) | 68  (0-750) |  | 78  (0-1557) | 7  (0-352) | 7  (0-183) | 108  (0-919) |
| ≥120 mIU/mL | 102  (0-1345) | 5  (0-67) | 20  (0-123) | 55  (0-255) |  | 102  (0-902) | 3  (0-16) | 6  (0-59) | 105  (0-495) |
| PRNT baseline categorized, *mean (range)*^£^ | N= (168,  45,18,39) | N= (121,  33,14,26) | N= (97,  30,11,27) | N= (91,  27,10,17) |  | N= (180,  68,27,41) | N= (126,  44,21,26) | N= (113,  41,18,32) | N= (104,  36,16,26) |
| 0-40 | 89  (0-2192) | 3  (0-50) | 51  (0-522) | 56  (0-529) |  | 76  (0-1557) | 7  (0-352) | 7  (0-183) | 112  (0-767) |
| 40-80 | 99  (0-1115) | 5  (0-90) | 32  (0-278) | 80  (0-508) |  | 69  (0-700) | 7  (0-186) | 7  (0-141) | 102  (0-919) |
| 80-120 | 107  (0-393) | 1  (0-8) | 13  (0-74) | 138  (0-750) |  | 118  (0-1043) | 3  (0-26) | 9  (0-101) | 96  (2-640) |
| >120 | 102  (0-1345) | 5  (0-67) | 20  (0-123) | 55  (0-254) |  | 102  (0-902) | 3  (0-16) | 6  (0-59) | 105  (1-495) |

**Supplementary Table 3:**  Descriptive results of the ELISpot T cell analysis with arithmetic means and ranges.

All descriptive results are based on maximum number of observations. Post int.: post-intervention sample 3-5 weeks after MMR/placebo at 5-7 months of age. Post routine: sample 3-5 weeks after routine MMR at 15 months of age. GMC: geometric mean count in SFU/10^6^ PBMCs (95% CI). N= (XX, YY) refers to XX individuals in first mentioned subgroup (e.g. Male) and YY individuals in other subgroup (e.g. Female).

^£^ Baseline PRNT results in the infants are missing for 1,18, 18 and 27 MMR donors and for 5, 20, 27 and 29 placebo donors, for mother, infant at baseline, infant post-int., and infant post-routine, respectively.

## Analytical results for ELISpot measles-proteome pools A-E

|  | Post intervention (MMR/placebo) | |  | Post routine MMR | |
| --- | --- | --- | --- | --- | --- |
|  | N^¤^ | GMR |  | N | GMR |
| *Measles proteome pools* | 134;143 | 12.3 (6.9-21.9) |  | 79;78 | 0.6 (0.3-0.9) |
| Pool A | 134;143 | 12.4 (6.6-23.6) |  | 79;78 | 0.5 (0.3-0.9) |
| Pool B | 134;143 | 13.5 (6.7-26.9) |  | 79;78 | 0.5 (0.3-0.9) |
| Pool C | 134;143 | 8.8 (4.4-17.6) |  | 79;78 | 0.6 (0.3-0.9) |
| Pool D | 134;143 | 6.7 (2.9-15.5) |  | 79;78 | 0.3 (0.2-0.6) |
| Pool E | 134;143 | 3.4 (1.7-6.9) |  | 79;78 | 0.5 (0.3-0.9) |

**Supplementary Table 4:** Analytical results of measles-specific T cells based on ELISpot measurements for the pools A-E from the measles proteome.

^¤^ XX; YY refers to X individuals in MMR group and Y individuals in placebo group. The pools contain a specific selection of the peptides from the measles proteome (see Methods section in the main text).

|  |  | Measles pools A-E censoring | | | | |
| --- | --- | --- | --- | --- | --- | --- |
|  |  | Post intervent.  < lower | | Post routine MMR  <lower | | |
|  |  | MMR | Placebo | | MMR | Placebo |
| *Analysis* |  |  |  | |  |  |
| Pool A |  | 62 | 126 | | 23 | 15 |
| Pool B |  | 67 | 130 | | 29 | 21 |
| Pool C |  | 74 | 126 | | 32 | 22 |
| Pool D |  | 100 | 134 | | 55 | 36 |
| Pool E |  | 99 | 126 | | 54 | 44 |

**Supplementary Table 5:** Censoring supporting the analytical results of measles-specific T cells based on ELISpot measurements for the pools A-E from the measles proteome (Suppl Table 4).

Censoring only occurred for the LLQ, as no well counts exceeded the ULQ at 150 SFU/well for neither the post intervention nor post routine MMR analyses.

## Full population, post-routine MMR results (all infants with a baseline and a post-routine MMR T cell analysis result, irrespective of whether a post-intervention analysis result was successfully obtained, or not)

|  | **Post routine MMR (MMR/placebo)** | |
| --- | --- | --- |
|  | N | GMR |
| Measles-specific T cells |  |  |
| GMR | 108;123 | 0.6 (0.4-0.9) |
| *Effect modification* |  |  |
| Sex |  |  |
| Male | 59;63 | 0.5 (0.2-0.9) |
| Female | 49;60 | 0.7 (0.4-1.4) |
| *Prematurity* |  |  |
| GA <37 | 11;6 | 2.0 (0.3-11.3) |
| GA ≥37 | 95;113 | 0.5 (0.3-0.9) |
| *Age at intervention* |  |  |
| < 6 months | 10;7 | 0.9 (0.2-4.9) |
| ≥ 6 months | 98;116 | 0.5 (0.1-1.9) |
| *Baseline PRNT* |  |  |
| < 120 mIU/mL | 93;102 | 0.6 (0.4-1.0) |
| ≥ 120 mIU/mL | 14;17 | 0.5 (0.2-1.4) |
| *Baseline PRNT* |  |  |
| < 40 mIU/mL | 63;69 | 0.5 (0.3-0.9) |
| 40-80 mIU/mL | 21;20 | 0.5 (0.2-1.2) |
| 80-120 mIU/mL | 9;13 | 0.5 (0.1-1.6) |
| ≥ 120 mIU/mL | 14;17 | 0.4 (0.2-1.2) |

**Supplementary Table 6:** Post-routine MMR analytical results based on the maximum number of observations when omitting the criterion regarding the presence of an ELISpot result at the post-intervention time point, thus it includes all infants having a T cell measurement at baseline and post-routine MMR. For the ELISpot, lower limit of detection is defined as 2 times the negative control well (left censoring). Upper limit of detection defined is defined as more than 150 spots in at least one of the wells (right censoring).

## Censoring table for full population post-routine MMR analytical results (supporting supplementary table 6)

|  |  | Post routine MMR | | | | |
| --- | --- | --- | --- | --- | --- | --- |
|  |  | < lower | | >upper | | |
|  |  | MMR | Placebo | | MMR | Placebo |
| *Analysis* |  |  |  | |  |  |
| Primary |  | 20 | 16 | | 2 | 4 |
| Sex, male |  | 12 | 10 | | 1 | 3 |
| Sex, female |  | 8 | 6 | | 1 | 1 |
| GA <37 |  | 3 | 2 | | 0 | 0 |
| GA ≥37 |  | 16 | 12 | | 2 | 4 |
| < 6 months |  | 1 | 0 | | 0 | 0 |
| ≥ 6 months |  | 19 | 16 | | 2 | 4 |
| PRNT dic: < 120 mIU/mL |  | 18 | 15 | | 2 | 3 |
| PRNT dic: ≥ 120 mIU/mL |  | 2 | 0 | | 0 | 1 |
| PRNT cat: < 40 mIU/mL |  | 11 | 7 | | 1 | 3 |
| PRNT cat: 40-80 mIU/mL |  | 4 | 6 | | 0 | 0 |
| PRNT cat: 80- 120 mIU/mL |  | 3 | 2 | | 1 | 0 |
| PRNT cat: ≥ 120 mIU/mL |  | 2 | 0 | | 0 | 1 |

**Supplementary Table 7:** The analytical results are calculated using a Tobit regression model (see main text for explanation). Here, the number of sample results which are either left-censored (having a result lower than the lowest limit of detection) or right-censored (having a result higher than the highest limit of detection) are presented for each of the analyses. This table corresponds to the results presented in Supplementary Table 6. For the ELISpot, the lower limit of detection is defined as 2 times the negative control well (left censoring). Upper limit of detection defined is defined as more than 150 spots in at least one of the wells (right censoring).

## Supplementary discussion on the effect of maternal antibodies

We examined whether the PRNT level at baseline reflecting maternal antibodies inversely affected cellular immune responses induced by early MMR vaccination. We found a tendency that lower PRNT levels at baseline were associated with higher ELISpot responses (PRNT < 120 mIU/ml had an ELSPOT GMR of 12.1 (95% CI: 6.5-22.6)) and vice versa (PRNT ≥ 120 mIU/ml had an ELISpot GMR of 4.6 (95% CI: 1.6-12.7)) (Table 2). A finer-grained analysis indicated that PRNT < 40 mIU/ml at baseline was associated with higher ELISpot response, and that the response dropped with increasing PRNT categorized as 40-80, 80-120 and ≥ 120 mIU/ml (Table 2). This tendency aligns well with the previously reported serological results, which also pointed at an association between maternally transferred antibodies and decreased vaccine-induced immune responses. Maternally transferred antibodies are a double-edged sword; they inhibit infection with measles virus but also block vaccination with live-attenuated measles vaccines. The timing of the first measles vaccine is delayed until the maternal antibodies have waned sufficiently to allow for proper take of the attenuated vaccine. The raison d'être of this study was that the current timing in Denmark of giving the first measles vaccine at 15 months of age is misaligned with the reality of the shorter period of protection provided by the reduced maternally transferred antibodies from the current generation of previously vaccinated mothers (PVMs) compared to the prior generation of previously infected mothers (PIMs). This potentially leaves infants unprotected against measles during the widened gap between the loss of maternal protection and the vaccine-induced acquisition of their own protection. As the gap widens, the infants are protected merely from herd immunity. Albeit an early MMR was less immunogenic than MMR after one year of age, it remains an important tool in the toolbox to prevent measles in the most vulnerable part of the population: the infants.

MMR vaccination was introduced in Denmark in 1987, 32 years prior to this trial. Since the average age of mothers giving birth in Denmark currently is about 31 years of age, this is an ideal time to conduct a trial like this, as one could expect an almost equal number of the mothers born before 1986 or after 1987 (Table 1). The former mothers are likely to have been naturally infected (i.e. PIMs), whereas the latter are likely to have been vaccinated (i.e. PVMs). We and others [2-4], have previously reported that these putative PIMs had significantly higher anti-measles antibodies and transferred more antibodies to their offspring than the putative PVMs did. Thus, in a Danish setting, examining infants from mothers born before 1986 with those born after 1987 is considered a proxy for examining infants of PIMs vs PVMs, and contributes to understanding whether moving the timing of the first MMR vaccination forward could reduce the time gap of missing maternal protection. Here, we examined whether mothers’ year of birth could be used as a proxy for whether early MMR vaccination induced a cellular immune response. As expected, our cellular analyses showed a tendency to increased cellular responses in infants born by PVMs vs. PIMs (Table 3) supporting our previous serological findings[3]. Combining these results, we provide evidence of humoral and cellular immunogenicity of early MMR especially in infants born by PVMs.

## References

1. Stryhn, A., et al., *A Systematic, Unbiased Mapping of CD8(+) and CD4(+) T Cell Epitopes in Yellow Fever Vaccinees.* Front Immunol, 2020. **11**: p. 1836.

2. Leuridan, E. and P. Van Damme, *Passive transmission and persistence of naturally acquired or vaccine-induced maternal antibodies against measles in newborns.* Vaccine, 2007. **25**(34): p. 6296-304.

3. Vittrup, D.M., et al., *Immunogenicity and reactogenicity following MMR vaccination in 5-7-month-old infants: a double-blind placebo-controlled randomized clinical trial in 6540 Danish infants.* EClinicalMedicine, 2024. **68**: p. 102421.

4. Waaijenborg, S., et al., *Waning of maternal antibodies against measles, mumps, rubella, and varicella in communities with contrasting vaccination coverage.* J Infect Dis, 2013. **208**(1): p. 10-6.
