# Supplementary material for: Measles-mumps-rubella-vaccination at 6 months of age induces measles-specific T cell responses: a randomized controlled trial
Source: Front Immunol. 2025 Mar 17;16:1546253. doi: 10.3389/fimmu.2025.1546253 (PMC11955646; doi:10.3389/fimmu.2025.1546253)
Supplement: Supplementary file 2 [file DataSheet2.docx]

Table of content

[Original statistical Analysis Plan for T-cell analyses in the Danish MMR trial 2](#_Toc183006185)

[Primary analysis 2](#_Toc183006186)

[Secondary analyses 2](#_Toc183006187)

[Exploratory analyses 3](#_Toc183006188)

[Sensitivity analyses 3](#_Toc183006189)

[Only in the early vaccinated population (MMR) without seroprotection at baseline 4](#_Toc183006190)

[Non-interventional, exploratory analyses 4](#_Toc183006191)

[Missing data 4](#_Toc183006192)

[Final Statistical Analysis Plan for T-cell analyses in the Danish MMR trial 5](#_Toc183006193)

[Primary analysis 5](#_Toc183006194)

[Secondary analyses 5](#_Toc183006195)

[Exploratory analyses 6](#_Toc183006196)

[Missing data 7](#_Toc183006197)

[Log for changes to the Statistical Analysis Plan for T-cell analyses in the Danish MMR trial 8](#_Toc183006198)

# Original statistical Analysis Plan for T-cell analyses in the Danish MMR trial

Measles-specific T-cell immunity after MMR at 5-7 months of age is a secondary outcome of the MMR trial.

The level of specific immunity against measles virus after MMR vaccination at 5-7 months of age will be measured in one way:

Interferon gamma-secreting T-cells when being stimulated with measles-epitopes by antigen-presenting cells.

The T-cell analyses are based on the ELIspot analysis for measles-specific T-cells. The measles proteome (isolate: [Moraten](https://www.ncbi.nlm.nih.gov/datasets/taxonomy/132484/)) was split into 5 pools of overlapping peptides (17 amino acids (aa) in length, stepping by 7 aa and overlapping by 10 aa), which together represents the entire Moraten proteome. Each sample was run as a duplicate to quadruplicate depending on the cell count in the sample. The total number of measles-specific T-cells is the sum of the median responses of each measles-proteome pool.

## Primary analysis

The endpoint of the primary analysis is the level of measles-specific T-cells as enumerated by ELIspot 21-34 days after MMR vaccination at 5-7 months of age.

The primary analysis will be conducted using Tobit regression with log-SFU as the outcome and randomization group as the exposure. The analysis will be adjusted for the baseline level of the outcome and two binary covariates, sex, and prematurity (gestational age < 37 weeks) since the randomization was stratified by these variables.

The estimate of interest is the geometric mean ratio (GMR) in spot-forming units (SFU) between the two randomization groups. This parameter will be obtained by exponentiating the estimated coefficient, $\hat{\beta}$_randomization_, corresponding to randomization group, and a 95% confidence interval of the GMR will be obtained by exponentiating the lower and upper confidence limits of the 95% confidence interval corresponding to $\hat{\beta}$_randomization_.

The proportion of infants in both groups who have measles-specific T-cells will be presented at all time points (count above lower limit of detection). This will be presented for mothers at baseline as well and according to their year of birth (before 1986, 1986-1987, after 1987) including further descriptive statistics.

*A T-cell responsiveness* is used to indicate a change in T-cell immunity status from a negative pre-vaccination to a positive level post-vaccination level. This will be assessed exploratorily. The lower limit of detection is defined as two times the negative control. An upper limit of detection is not sharply defined, but levels above 150 spots per well in the assay may be difficult to distinguish depending on spot size. Thus, if and only if the (median) count for at least one of the (five) wells surpasses 150, the record will be right censored using the reported SFU value as the upper limit for the record.

## Secondary analyses

The secondary analyses presented below follow the same structure (Tobit regression with the stated covariates) as that of the primary analysis described above. The same holds for the exploratory analyses which are described in the next subsection.

The mother’s year of birth is used as a proxy for measles immunization status. Individuals born before 1986 are likely to have been infected with wild-type measles, whereas individuals born after the introduction of MMR in 1987 are likely to have been vaccinated and never wild-type measles exposed (WT infected). For individuals born between 1986-1987, it is impossible to deduce whether they were WT-infected or not, and thus, were assigned to an intermediate group consisting of WT-infected and vaccinated individuals.

The planned secondary analyses of specific immunity around 1 month after randomization are summarized in the table below:

Data regarding measles-neutralizing antibody levels measured by plaque reduction neutralization test (PRNT) is taken into consideration as the dichotomized seroprotection (seroprotective cutoff > 120 mIU/mL) and as a categorized level (<40, 40-80, 80-120, >120).

| **Measure** | **Time point** | **Effect modification by** |
| --- | --- | --- |
| specific T cell | Post-intervention | Sex |
| specific T cell | Post-intervention | Gestational age < 37 weeks |
| specific T cell | Post-intervention | Age at vaccination < 6 months |
| specific T cell | Post-intervention | Mother immunization status (proxy: year of birth) |
| specific T cell | Post-intervention | PRNT baseline dichotomized |
| specific T cell | Post-intervention | PRNT baseline categorized |
| specific T cell | Post-routine MMR |  |

## Exploratory analyses

The planned exploratory analyses of specific immunity are summarized in the table below:

| **Measure** | **Time point** | **Effect modification by** |
| --- | --- | --- |
| specific T cell | Post-routine MMR | Sex |
| specific T cell | Post-routine MMR | Gestational age < 37 weeks |
| specific T cell | Post-routine MMR | Age at vaccination < 6 months |
| specific T cell | Post-routine MMR | Mother immunization status (proxy: year of birth) |
| specific T cell | Post-routine MMR | Mother T-cell level |
| specific T cell | Post-routine MMR | PRNT baseline dichotomized |
| specific T cell | Post-routine MMR | PRNT baseline categorized |
| specific T cell, measles pools 1-5 separately | Post-intervention and post-routine MMR |  |

## Sensitivity analyses

| **Measure** | **Time point** | **Effect modification by** |
| --- | --- | --- |
| specific T cell | Post-intervention | Sensitivity analysis regarding full data set, including those that did not pass the > 2 x background signal (SEB/CD3-stimulation). |
| specific T cell | Post-routine MMR | Sensitivity analysis regarding full data set, including those that did not pass the > 2 x background signal (SEB/CD3-stimulation). |

## Only in the early vaccinated population (MMR) without seroprotection at baseline

| **Measure** | **Time point** | **Comparison of interest** |
| --- | --- | --- |
| specific T cell | Post-routine MMR | Seroprotection status measured in PRNT at post-intervention |
| specific T cell | Post-routine MMR | Post-intervention T cell level analyzed as a continuous variable using splines |

## Non-interventional, exploratory analyses

| **Measure** | **Time point** | **Effect modification by** |
| --- | --- | --- |
| specific T cell | Post-routine MMR | T-cell post-intervention |
| specific T cell | Post-routine MMR | PRNT post-intervention dichotomized |
| specific T cell | Post-routine MMR | PRNT post-intervention categorized |

The relationship between maternal specific T cell SFU count, and the response of the infant will be assessed. Also, the relationship between the PRNT and T-cell measurements will be explored.

## Missing data

The T-cell studies are exploratory outcomes in the overall MMR trial. The power calculation estimated a total of 500 complete mother-child-sets (child pre-randomization, child post-randomization, child post-routine exposure, and mother pre-randomization) as sufficient for the serology studies. We prioritized the main outcome, the PRNT post-intervention analysis, and thus the T-cell samples were not as numerous as the serology samples. This was further complicated by the fact, that a rather large blood volume was needed for the T-cell assay to function, and sampling in infants is a delicate situation, thus, unsuccessful sampling regarding the T-cell blood volume was a premise. Non-complete sets include sets with any missing samples from child baseline, child post-randomization, child post-routine exposure, or mother, but regarding the primary analysis, only samples regarding child at baseline and post-randomization are needed. Statistical analyses defined in the present document will be performed on the analysis sets, defined by non-missing T-cell data at baseline and at post-randomization criterion.

For an ELIspot result to be perceived as valid, the positive control measured as T-cell stimulation by SEB/CD3 needs to be a response two times higher than the negative control. Non-valid analysis results were more frequent in the baseline and post-intervention samples than in mother and post-routine samples. Thus, the lab analysis results are not thought to be missing completely at random.

# Final Statistical Analysis Plan for T-cell analyses in the Danish MMR trial

Measles-specific T-cell immunity after MMR at 5-7 months of age is a secondary outcome of the MMR trial.

The level of specific immunity against measles virus after MMR vaccination at 5-7 months of age will be measured in one way:

Interferon gamma-secreting T-cells when being stimulated with measles-epitopes by antigen-presenting cells.

The T-cell analyses are based on the ELIspot analysis for measles-specific T-cells. The measles proteome (isolate: [Moraten](https://www.ncbi.nlm.nih.gov/datasets/taxonomy/132484/)) was split into 5 pools of overlapping peptides (17 amino acids (aa) in length, stepping by 7 aa and overlapping by 10 aa), which together represent the entire Moraten strain proteome. Each sample was run as a duplicate to quadruplicate depending on the cell count in the sample. The total number of measles-specific T-cells is the sum of the median responses of each measles-proteome pool.

## Primary analysis

The endpoint of the primary analysis is the level of measles-specific T-cells as enumerated by ELIspot 21-34 days after MMR vaccination at 5-7 months of age.

The primary analysis will be conducted using Tobit regression with log-SFU (spot-forming units) as the outcome and randomization group as the exposure. The analysis will be adjusted for the baseline level of the outcome and two binary covariates, sex, and prematurity (gestational age < 37 weeks) since the randomization was stratified by these variables.

The estimate of interest is the geometric mean ratio (GMR) in SFU between the two randomization groups. This parameter will be obtained by exponentiating the estimated coefficient, $\hat{\beta}$_randomization_, corresponding to randomization group, and a 95% confidence interval of the GMR will be obtained by exponentiating the lower and upper confidence limits of the 95% confidence interval corresponding to $\hat{\beta}$_randomization_.

The proportion of infants in both groups who have measles-specific T-cells will be presented at all time points (count above lower limit of detection). This will be presented for mothers at baseline as well and according to their year of birth (before 1986, 1986-1987, after 1987) including further descriptive statistics.

*A T-cell responsiveness* is used to indicate a change in T-cell immunity status from a negative pre-vaccination to a positive level post-vaccination level. This will be assessed exploratorily. The lower limit of detection is defined as two times the negative control at plate level in either the positive control (SEB/CD3) or in either measles pool. An upper limit of detection is not sharply defined, but levels above 150 spots per well in the assay may be difficult to distinguish depending on spot size. Thus, if and only if the (median) count for at least one of the (five) pools surpasses 150, the record will be right-censored using the reported SFU value (i.e., the sum of the five pools) as the upper limit for the record.

## Secondary analyses

The secondary analyses presented below follow the same structure (Tobit regression with the stated covariates) as that of the primary analysis described above. The same holds for the exploratory analyses which are described in the next subsection.

The mother’s year of birth is used as a proxy for measles immunization status. Individuals born before 1986 are likely to have been infected with wild-type measles, whereas individuals born after the introduction of MMR in 1987 are likely to have been vaccinated and never wild-type measles exposed (WT infected). For individuals born between 1986-1987, it is impossible to deduce whether they were WT-infected or not, and thus, were assigned to an intermediate group consisting of WT-infected and vaccinated individuals.

The planned secondary analyses of specific immunity around 1 month after randomization are summarized in the table below:

Data regarding measles-neutralizing antibody levels measured by plaque reduction neutralization test (PRNT) is taken into consideration as the dichotomized seroprotection (seroprotective cutoff > 120 mIU/mL) and as a categorized level (<40, 40-80, 80-120, >120).

| **Measure** | **Time point** | **Effect modification by** |
| --- | --- | --- |
| specific T cell | Post-intervention | Sex |
| specific T cell | Post-intervention | Gestational age < 37 weeks |
| specific T cell | Post-intervention | Age at vaccination < 6 months |
| specific T cell | Post-intervention | Mother immunization status (proxy: year of birth) |
| specific T cell | Post-intervention | PRNT baseline dichotomized |
| specific T cell | Post-intervention | PRNT baseline categorized |
| specific T cell | Post-routine MMR |  |

## Exploratory analyses

The planned exploratory analyses of specific immunity are summarized in the table below:

| **Measure** | **Time point** | **Effect modification by** |
| --- | --- | --- |
| specific T cell | Post-routine MMR | Sex |
| specific T cell | Post-routine MMR | Gestational age < 37 weeks |
| specific T cell | Post-routine MMR | Age at vaccination < 6 months |
| specific T cell | Post-routine MMR | Mother immunization status (proxy: year of birth) |
| specific T cell | Post-routine MMR | PRNT baseline dichotomized |
| specific T cell | Post-routine MMR | PRNT baseline categorized |
| specific T cell, measles pools 1-5 separately | Post-intervention and post-routine MMR |  |

The relationship between the PRNT and T-cell measurements will be explored. Specifically, the relationship will be described based on seroconversion (SCR) and cellular conversion (CCR) rates and a joint immune conversion rate (ICR) reflecting a measles-specific immune response, whether serological (SCR), cellular (CCR) or both.

- SCR is defined as the proportion of individuals with either a four-fold increase in concentration of neutralizing antibodies from pre- to post-exposure level (intervention (MMR/placebo) or routine MMR) or a change in status from unprotected to protected based on the conventional cutoff at 120 mIU/mL.
- CCR is defined as the proportion of individuals with measles-specific T cell responses exceeding the 95% percentile observed in the overall infant population at baseline, which is expected to be measles naïve.

ICR is defined as the proportion of individuals achieving SCR and/or CCR from pre- to post-exposure (intervention (MMR/placebo) or routine MMR).

## Missing data

The T-cell studies are exploratory outcomes in the overall MMR trial. The power calculation estimated a total of 500 complete mother-child pairs (child pre-randomization, child post-randomization, child post-routine exposure, and mother pre-randomization) as sufficient for the serology studies. We prioritized the main outcome, the PRNT post-intervention analysis, and thus the T-cell samples were not as numerous as the serology samples. This was further complicated by the fact, that a rather large blood volume was needed for the T-cell assay to function, and sampling in infants is a delicate situation, thus, unsuccessful sampling regarding the T-cell blood volume was a premise. Non-complete sets include sets with any missing sample from child baseline, child post-randomization, child post-routine exposure, or mother, but regarding the primary analysis, only samples regarding child at baseline and post-randomization are needed. Statistical analyses defined in the present document will be performed on the analysis sets, defined by non-missing T-cell data at baseline and at post-randomization criterion.

For an ELISpot result to be perceived as valid, the positive control measured as T cell stimulation by SEB/CD3 or in the measles pools needs to be a response two times higher than the negative control at plate level. Non-valid analysis results were omitted at laboratory analysis level as the setup was considered flawed if the above-mentioned criteria were not met. The lab analysis results are thought to be missing completely at random since the samples from different sampling time points (mother, child pre- and post-intervention and post-routine MMR were run together).

# Log for changes to the Statistical Analysis Plan for T-cell analyses in the Danish MMR trial

**Changes implemented January 24, 2024**

1. The lower limit of detection needed further specification both in the section “Primary analysis” and “Missing data”. Specifically, the ELIspot results are perceived valid if the plate setup produced responses two times higher than the negative control in either the positive control (SEB/CD3 activation) or in the measles pools.

The criterion was changed as young infant T cells are not as responsive to the SEB/CD3 activators as more mature T cells. Thus, a response in the positive control well with only 10,000 T cells cannot be expected to produce a countable response in young infants. However, when the plate works, we expect the plate to count responses if any, and thus, a response equal to 0 is also valid.

1. The sensitivity analyses table was omitted as the requirement for valid samples was changed according to the above-mentioned description.
2. In the “Missing data section”, the sentence “Thus, the lab analysis results are not thought to be missing completely at random.“ was changed to “The lab analysis results are thought to be missing completely at random since the samples from different sampling time points (mother, child pre- and post-intervention and post-routine MMR were run together).” as plate validity was deterministic for sample validity (see first comment in changes to SAP).

**Changes implemented August 20, 2024**

1. The analysis regarding effect modification of the mother’s T cell level on the post routine MMR specific T cell level was omitted along with the sentence: “The relationship between maternal specific T cell SFU count, and the response of the infant will be assessed.”.
2. A definition and a description of SCR, CCR, and ICR has been specified.
3. Non-interventional, exploratory analyses regarding effects of post-intervention results on post-routine MMR T cell counts were omitted.
